# Fetal mitochondrial DNA in maternal plasma in surrogate pregnancies: Detection and topology

**DOI:** 10.1002/pd.5860

**Published:** 2020-11-12

**Authors:** Mary‐Jane L. Ma, Sergey Yakovenko, Haiqiang Zhang, Suk Hang Cheng, Valentina Apryshko, Alex Zhavoronkov, Peiyong Jiang, K. C. Allen Chan, Rossa W. K. Chiu, Y. M. Dennis Lo

**Affiliations:** ^1^ Li Ka Shing Institute of Health Sciences The Chinese University of Hong Kong Hong Kong China; ^2^ Department of Chemical Pathology The Chinese University of Hong Kong, Prince of Wales Hospital Hong Kong China; ^3^ Altravita IVF‐clinic ECO Center Moscow Russia; ^4^ Biophysics Department, Faculty of Physics Moscow State University Moscow Russia; ^5^ Insilico Medicine Ltd Hong Kong Science and Technology Park Hong Kong China

## Abstract

**Objectives:**

Due to the maternally‐inherited nature of mitochondrial DNA (mtDNA), there is a lack of information regarding fetal mtDNA in the plasma of pregnant women. We aim to explore the presence and topologic forms of circulating fetal and maternal mtDNA molecules in surrogate pregnancies.

**Methods:**

Genotypic differences between fetal and surrogate maternal mtDNA were used to identify the fetal and maternal mtDNA molecules in plasma. Plasma samples were obtained from the surrogate pregnant mothers. Using cleavage‐end signatures of *Bfa*I restriction enzyme, linear and circular mtDNA molecules in maternal plasma could be differentiated.

**Results:**

Fetal‐derived mtDNA molecules were mainly linear (median: 88%; range: 80%–96%), whereas approximately half of the maternal‐derived mtDNA molecules were circular (median: 51%; range: 42%–60%). The fetal DNA fraction of linear mtDNA was lower (median absolute difference: 9.8%; range: 1.1%–27%) than that of nuclear DNA (median: 20%; range: 9.7%–35%). The fetal‐derived linear mtDNA molecules were shorter than the maternal‐derived ones.

**Conclusion:**

Fetal mtDNA is present in maternal plasma, and consists mainly of linear molecules. Surrogate pregnancies represent a valuable clinical scenario for exploring the biology and potential clinical applications of circulating mtDNA, for example, for pregnancies conceived following mitochondrial replacement therapy.

**What is already known about this topic?**

- In natural, non‐surrogate pregnancies, fetal nuclear DNA in maternal plasma consists of short linear DNA fragments that have a shorter size distribution than the maternal background nuclear DNA.
- Both linear and circular mitochondrial DNA (mtDNA) molecules co‐exist in plasma of liver and bone marrow transplant patients.
- Liver tissues contribute a considerable amount of mtDNA predominantly in a linear form to the plasma DNA pool.
- mtDNA molecules of hematopoietic origin are mainly in a circular form in plasma.

**What does this study add?**

- Fetal and maternal mtDNA molecules could be distinguished in the plasma of surrogate pregnant mothers.
- Linear and circular mtDNA molecules co‐existed in the plasma of surrogate pregnant women.
- The fetal mtDNA molecules in surrogate maternal plasma appeared to be predominantly in a linear form, while approximately half of the surrogate maternal mtDNA molecules were in a circular form.
- The fetal mtDNA fraction was less abundant than the fetal nuclear DNA fraction in surrogate pregnant women.
- Circulating fetal mtDNA molecules consisted of more short molecules compared with the surrogate maternal mtDNA molecules.

## 1 INTRODUCTION

Most studies of cell‐free DNA in plasma focus on DNA molecules in a linear form derived from the nucleus. These linear plasma DNA molecules display characteristic fragmentation patterns, and are associated with nucleosomal structures.^1^ For instance, the modal frequency of fragment sizes is approximately 166 bp, accompanied by multiple 10‐bp periodic peaks of small sizes. Such characteristic patterns have inspired recent interest in plasma DNA fragmentomics, encompassing areas such as plasma DNA preferred ends,^2^, ^3^ fragment sizes,^1^, ^4^, ^5^ end motifs,^6^, ^7^ nucleosome footprints,^8^, ^9^, ^10^ plasma DNA end orientations^11^ as well as biological links between fragmentation processes and nucleases.^6^, ^12^, ^13^

In addition to studying the properties of nuclear DNA molecules in plasma, more recently, on the study of subjects following liver and bone marrow transplantation, we have demonstrated that there are two different topologic forms of mitochondrial DNA (mtDNA), namely linear and circular forms, present in plasma DNA.^14^ Of note, mtDNA molecules of hematopoietic origin appear to be mainly circular, in sharp contrast to the liver‐derived mtDNA molecules that mainly display a linear form.^14^ These results suggest that plasma mtDNA topology might vary depending of the tissues of origin of the detected mtDNA.

Topologic study of plasma DNA can also be extended to autosomal DNA molecules.^15^ For example, the presence of extrachromosomal circular DNA (eccDNA) was observed in both maternal and fetal DNA molecules in pregnant women, and displaying distinct size patterns.^15^ While circular mtDNA and eccDNA in plasma are governed by different biologic mechanisms, our work has highlighted their commonality in their accessibility to being studied using similar methodologies.

In this study, we explore whether tissue‐associated topologic forms of mtDNA might be present in the plasma of pregnant women. Pregnancy offers an attractive model for studying the biology of tissue‐specific plasma DNA molecules because there is the co‐existence of fetal‐ and maternal‐derived DNA in maternal plasma.^16^ However, the study of fetal mtDNA in maternal plasma is complicated by the fact that mitochondria are inherited from the mother. Hence, it would not normally be possible to differentiate fetal and maternal mtDNA molecules in natural pregnancies. In the context of this study, a surrogate mother is a woman engaged by a couple to become pregnant with an embryo fertilized using gametes of the couple. Thus, in this study, surrogate pregnancy was chosen as a model to explore the topologic forms of circulating fetal mtDNA.

## 2 METHODS AND MATERIALS

### 2.1 Subjects

The subject recruitment and sample collection were conducted in Altravita IVF‐clinic, ECO Center, Moscow, Russia. Five surrogate pregnant cases were recruited. Biological parents involved in the five surrogate pregnancies gave informed written consent. Informed verbal consent was obtained from the five surrogate pregnant women. Plasma and buffy coat samples of surrogate pregnant subjects were collected during the second or third trimester (21–31 weeks). Buffy coat or saliva samples from the oocyte donor subjects were collected. Review of the clinical research protocol was conducted and approved by the ethics committee of the ECO Center, Moscow, Russia (reference number: Protocol No. 3). The Joint Chinese University of Hong Kong – New Territories East Cluster Clinical Research Ethics Committee approved (reference number: 2019.568) the data generation and data analysis of the study.

### 2.2 DNA extraction and sequencing library preparation

Peripheral blood samples of the surrogate pregnant women were collected into EDTA‐containing tubes and centrifuged at 1600 *g* at 4°C for 10 min. The upper portion of plasma was recentrifuged at 16,000 g at 4°C for 10 min to obtain cell‐free plasma. Plasma DNA was extracted from 3–4 ml of plasma using the QIAamp Circulating Nucleic Acid Kit (Qiagen). Half of the plasma DNA was digested by the restriction enzyme *Bfa*I (New England Biolabs) while the other half of the plasma DNA was processed without the restriction enzyme digestion step. Ten units of *Bfa*I (restriction recognition site: C^TAG; ^ denoting the cutting site) were used in the cleavage of 20 ng plasma DNA at 37°C for 120 min. Tissue DNA samples of buffy coat or saliva from the oocyte donors and buffy coat from the surrogate mothers were extracted using the QIAamp DNA Mini and Blood Mini Kit. The S220 Focused‐ultrasonicator (Covaris) was used to shear tissue DNA (from both surrogate mothers and oocyte donor mothers) into sizes of around 200 bp.

DNA libraries were generated by the TruSeq Nano DNA Library Prep Kit (Illumina) and ligated with xGEN Dual Index UMI adapters (IDT). A unique dual‐index sequence is a sequence tag that recognizes a sample identity for sample multiplexing purpose. Unique molecular identifier (UMI) is a technology for ascertaining molecular identity by adding a unique sequence tag together with an adaptor to the original DNA template before PCR amplification and sequencing. The size and concentration of DNA libraries were then analyzed respectively by 2100 TapeStation (Agilent) and Qubit 3.0 Fluorometer (Thermo Fisher Scientific).

### 2.3 Hybridization‐based target enrichment

SeqCap EZ Prime Developer Probes (Roche) targeting human mtDNA were used in the target‐capture enrichment procedures after the DNA library preparation. The NimbleGen SeqCap Hybridization and Wash Kit (Roche) was used during capture procedures. The 4200 TapeStation (Agilent) and the Qubit 3.0 Fluorometer (Thermo Fisher Scientific) were used to evaluate the quality of the target‐captured DNA libraries. Targeted regions of interest included 1000 SNP (single nucleotide polymorphism) sites and human mtDNA regions for probe design and synthesis (Roche NimbleGen Inc.) as previously described.^14^

### 2.4 Sequencing and alignment

Target‐captured DNA libraries were sequenced in a 2 × 70 bp paired‐end mode on a NextSeq 500 system (Illumina) using the NextSeq 500 High Output Reagent Cartridge v2 Kit (Illumina). Sequencing reads were attributed to multiple samples based on unique dual‐index sequences using Picard tools (https://broadinstitute.github.io/picard/). The demultiplexed reads were aligned to the reference genome database including the human reference genome (NCBI37/hg19) and the mitochondrial genome using BWA.^17^ Reads with UMIs information were used to generate consensus reads using fgbio tools.^18^ SOAP2^19^ was utilized to realign the consensus reads to determine the genomic origins for each paired‐end sequenced reads. During the alignment based on SOAP2,^19^ we allowed up to two mismatches to make those reads with the single nucleotide variants mappable for the downstream analysis. The fetal and maternal DNA molecules were differentiated from the mapped reads based on informative single nucleotide variants.^1^

### 2.5 Determination of linear and circular mtDNA

**FIGURE 1:**
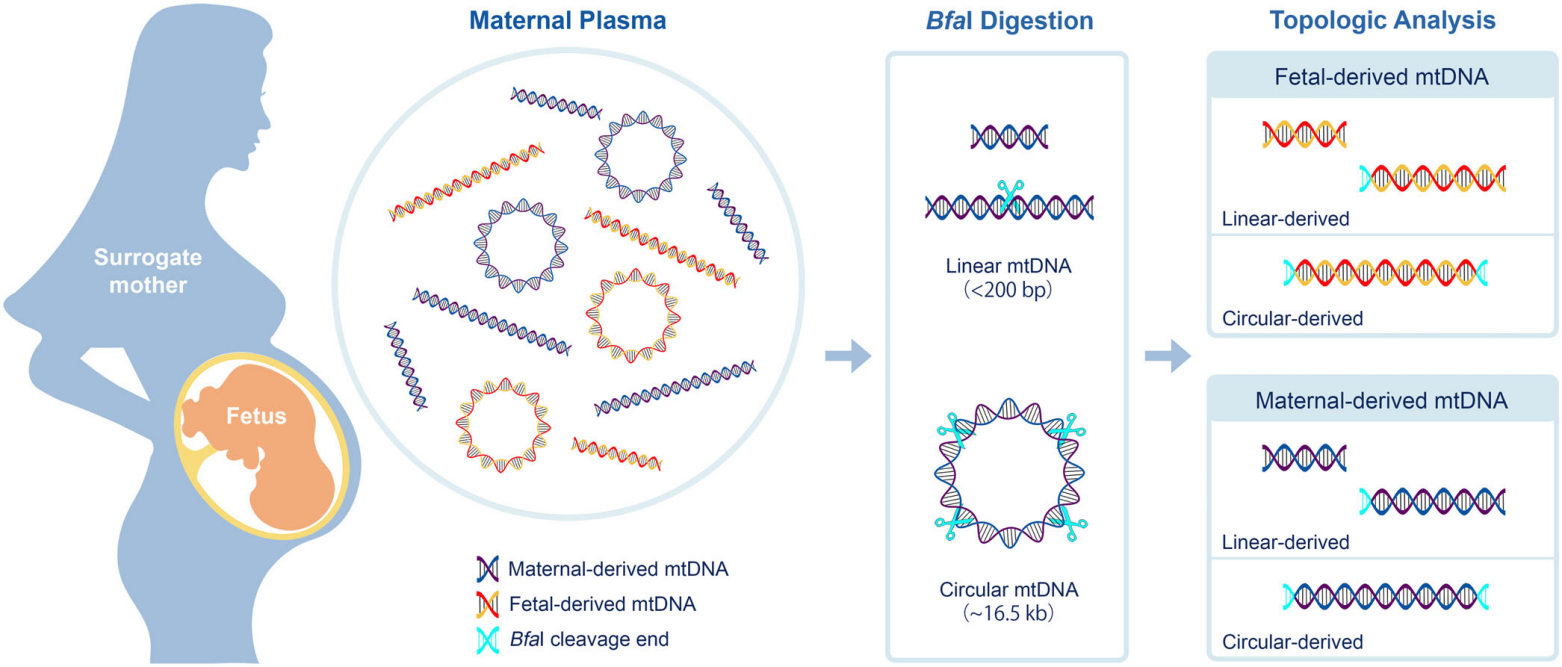
Schematic illustration of the topological assessment of mtDNA in surrogate pregnancies. Fetal mtDNA may be released into surrogate maternal plasma through the placenta during pregnancy. *Bfa*I cleavage signatures were used for differentiating the intact circular mtDNA molecules (~16.5 kb) from pre‐existing linear mtDNA molecules (generally <200 bp). *Bfa*I has a 4‐bp recognition sequence which by random chance should occur once every 256 bp (1/44). Therefore, fragments originated from intact circular mtDNA molecules would carry two *Bfa*I‐cleavage ends (Cyan DNA) after *Bfa*I digestion (Cyan scissor), whereas the majority of the fragments derived from pre‐existing linear mtDNA molecules would have no more than one cleavage end (Cyan DNA). Single nucleotide variants in mitochondrial genomes were used to determine the physical forms of fetal and maternal mtDNA in plasma of surrogate pregnant women. Combined with topological assessment of mtDNA, we could identify if the fetal‐derived (red‐yellow DNA) or maternal‐derived (purple‐blue DNA) mtDNA molecules were in a circular or linear form according to the patterns of *Bfa*I‐cleavage signatures [Colour figure can be viewed at wileyonlinelibrary.com]

The restriction enzyme *Bfa*I was used for differentiating linear and circular mtDNA as previously described.^14^ This enzyme cleaves C^TAG sites and is expected to cut DNA once every 256 bp (1/4^4^). Briefly, after plasma DNA was treated with *Bfa*I, the linear mtDNA molecules in plasma would mainly either remain uncleaved or be cleaved into two fragments both with one *Bfa*I‐cleaved end signature. On the contrary, an intact circular mtDNA (~16.5 kb), containing a total of 98 *Bfa*I cutting sites, would be cleaved into linear mtDNA fragments each bearing two cleavage ends. The principle of the topologic analysis of plasma mtDNA for surrogate pregnant subjects is illustrated in Figure 1.

### 2.6 Determination of informative variants in mitochondrial genomes and nuclear genomes

The genotypes of fetal and surrogate mother's mitochondrial genomes were determined by sequencing relevant tissue DNA including buffy coat or saliva DNA from oocyte donors or surrogate mother's buffy coat DNA. A cell can contain numerous copies of mtDNA among which some may carry different variants in certain locations of the mtDNA genome, but some may not, which is referred to as mtDNA heteroplasmy. To minimize the influence of the mtDNA heteroplasmy in a tissue, we focused on the informative variants in the mitochondrial genome for which the alleles of a single nucleotide variant site were identical (i.e., homoplasmic) but different between the surrogate mother and oocyte donor. Due to the maternal inheritance of mtDNA, fetal‐specific mtDNA variants were represented by oocyte donor specific mtDNA variants. Fetal mtDNA fraction (*F*_*m*_) was deduced by the allelic ratio between fetal‐specific variants (e.g., *A*) and surrogate maternal specific variants (e.g., *B*) present in the mitochondrial genome:

$$F_{m}=\frac{A}{A+B}\times 100\%.$$

In our present study, the placental tissues were not available and the fetal genotypes were determined non‐invasively. Thus, we could not directly obtain the fetal genotype information. In our analysis, we utilized the nuclear genotypes from tissue genomic DNA from the surrogate mother and the oocyte donor mother, in conjugation with sequencing result from one plasma aliquot, to deduce the informative SNPs allowing the fetal nuclear DNA fraction (*F*_*n*_) estimation in the second plasma aliquot. For the sake of simplicity, we first focused on the SNPs which were homozygous for the same allele both in the surrogate mother (AA) and the oocyte donor mother (AA). Among those SNPs, if a locus showed a different allele in one plasma aliquot, such allele would likely be derived from the fetus. Due to our bioinformatics algorithm, the fetal SNPs used in the fetal nuclear DNA fraction calculation would be heterozygous (AB) in the fetus:

$$F_{n}=\frac{2B}{A+B}\times 100\%.$$

### 2.7 Statistical analysis

Sequencing data analysis was performed using in‐house bioinformatics programs written in Perl and R languages. A *p*‐value of <0.05 was considered as statistically significant with all two‐tailed probabilities.

## 3 RESULTS

### 3.1 *Bfa*I enzymatic digestion for maternal plasma DNA of surrogate pregnant women

**FIGURE 2:**
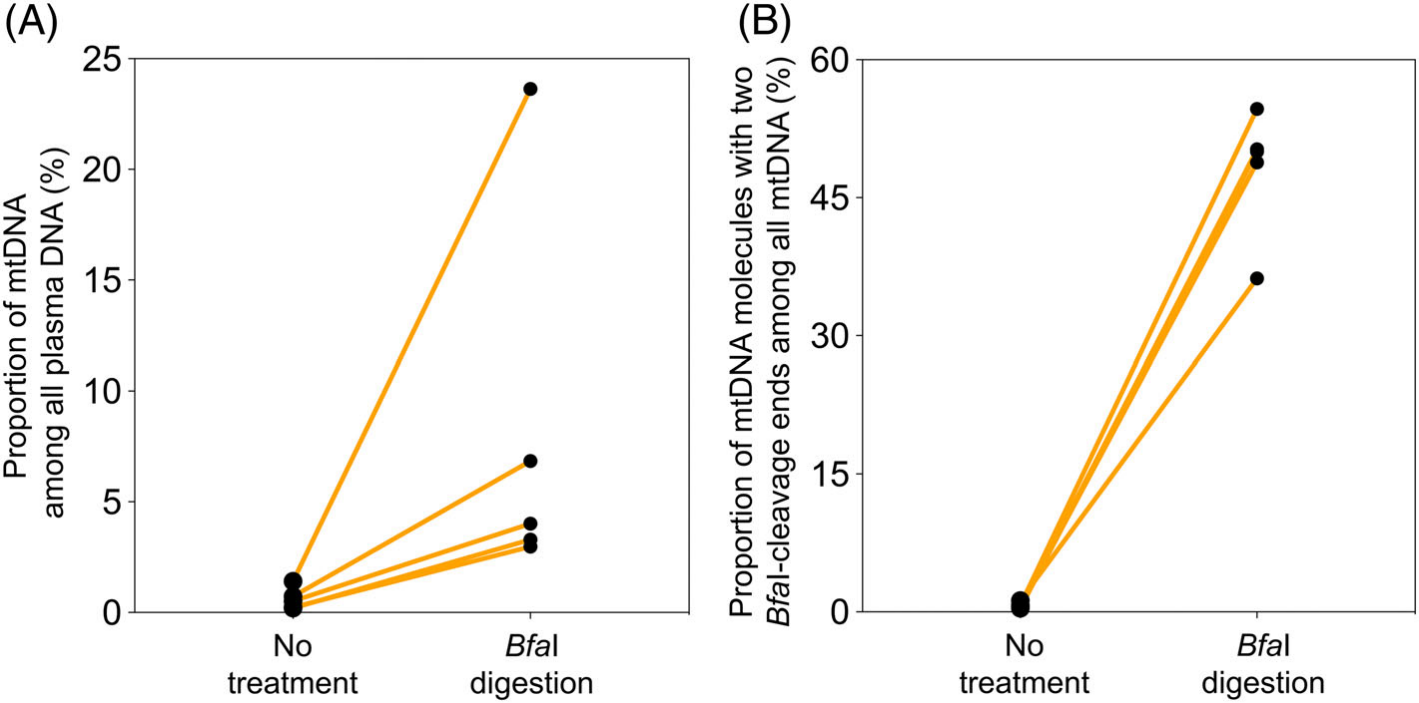
Effects of *Bfa*I cleavage on plasma mtDNA. (A) Comparison of the proportion of mtDNA among all DNA in plasma of surrogate pregnant women with and without *Bfa*I digestion. (B) Comparison of the proportion of mtDNA molecules with two *Bfa*I‐cleavage ends (circular‐derived mtDNA) among all mtDNA in plasma of surrogate pregnant women with and without *Bfa*I digestion [Colour figure can be viewed at wileyonlinelibrary.com]

We obtained a median on‐target depth of 1005× (range: 529–1467×) and 1081× (range: 693–2106×) for samples with and without *Bfa*I digestion, respectively. There was a 7.9‐fold increase in the median proportion of mtDNA among all plasma DNA increased in plasma DNA samples with *Bfa*I digestion (median: 4.0%; range: 3.0%–24%), compared with those without digestion (median: 0.51%; range: 0.2–1.4%) (*p*‐value: 0.004, Mann–Whitney *U* test) (Figure 2(A)). The median proportion of circular mtDNA fragments (i.e., for those carrying two enzymatic cleavage ends) among all mtDNA molecules in *Bfa*I digested samples was 50% (range: 36–55%), which was a 76‐fold increase compared with those without digestion (median: 0.66%; range: 0.41–1.2%) (*p*‐value = 0.004, Mann–Whitney *U* test) (Figure 2(B)). Overall, these results suggested the existence of circular mtDNA in plasma DNA of surrogate pregnant subjects.

As shown in Figure S1A, in contrast to plasma DNA of surrogate pregnant women without *Bfa*I treatment exhibiting a relatively smooth size distribution of mtDNA peaked at around 60 bp, plasma DNA with *Bfa*I digestion displayed an irregular distribution with a series of sporadic spikes up to 550 bp. The results were largely consistent with what was previously observed in plasma DNA of liver transplant patients.^14^ However, we did not observe such differences in the size distribution of nuclear DNA molecules between before and after *Bfa*I digestion (Figure S1B). The data provided further evidence that there may exist circular mtDNA in plasma of surrogate pregnant women.

### 3.2 Topologic analysis of fetal DNA and maternal mtDNA

We identified single nucleotide variants in the mitochondrial genome whose sequences differed between oocyte donor and paired surrogate maternal tissues, termed informative variants. To get fetal genotype information, buffy coat or saliva DNA samples from oocyte donors were sequenced to a median on‐target depth of 1636× (939–2283×). The matched buffy coat samples of surrogate mothers (5 pairs) were sequenced to a median on‐target depth of 1575× (interquartile range: 618–2361×). We obtained a median of 29 informative variants (range: 13–46) in the mitochondrial genome and 674 informative SNPs in the nuclear genome (range: 469–836, out of 1000 SNPs designed for target capture). The molecules carrying fetal‐specific mitochondrial variants (fetal‐derived mtDNA) and surrogate maternal‐specific mitochondrial variants were determined. We identified a median of 1176 (range: 260–2864) fetal‐specific mtDNA molecules and a median of 49,623 (range: 13,227–214,898) surrogate maternal‐specific mtDNA molecules among five maternal plasma samples.

**FIGURE 3:**
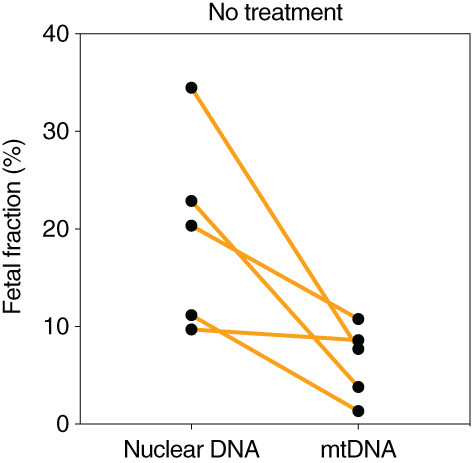
Fetal DNA fraction in nuclear DNA and mtDNA among surrogate maternal plasma DNA samples without *Bfa* I treatment [Colour figure can be viewed at wileyonlinelibrary.com]

For the maternal plasma DNA samples without *Bfa*I digestion, the median fetal nuclear DNA fraction was 20% (range: 9.7%–35%), whereas the fetal mtDNA fraction was found to be much lower (median: 7.8%; range: 1.4%–11%) (*p*‐value = 0.008, Mann–Whitney *U* test) (Figure 3). No significant correlation was observed between the fetal fractions of circulating nuclear DNA and mtDNA (r = 0.23; *p*‐value = 0.71).

**FIGURE 4:**
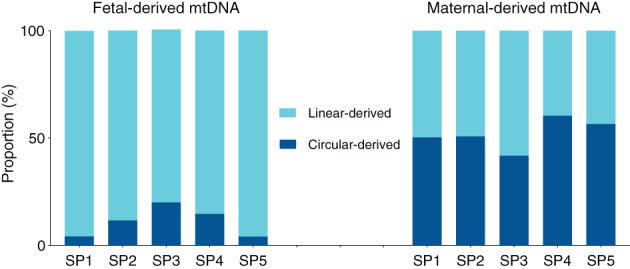
The proportions of linear and circular forms in fetal‐ and maternal‐derived mtDNA in plasma across five surrogate pregnancies (SP1 to SP5) [Colour figure can be viewed at wileyonlinelibrary.com]

On the basis of *Bfa*I cleavage end signature analysis, we found that the proportion of linear mtDNA among fetal‐derived mtDNA molecules (i.e., placenta‐derived; median: 88%; range: 80%–96%) was much higher than that among maternal‐derived mtDNA molecules (i.e., mainly of hematopoietic and liver origin; median: 49%; range: 40%–58%) (Figure 4).

### 3.3 Size distribution of fetal‐ and maternal‐derived mtDNA

**FIGURE 5:**
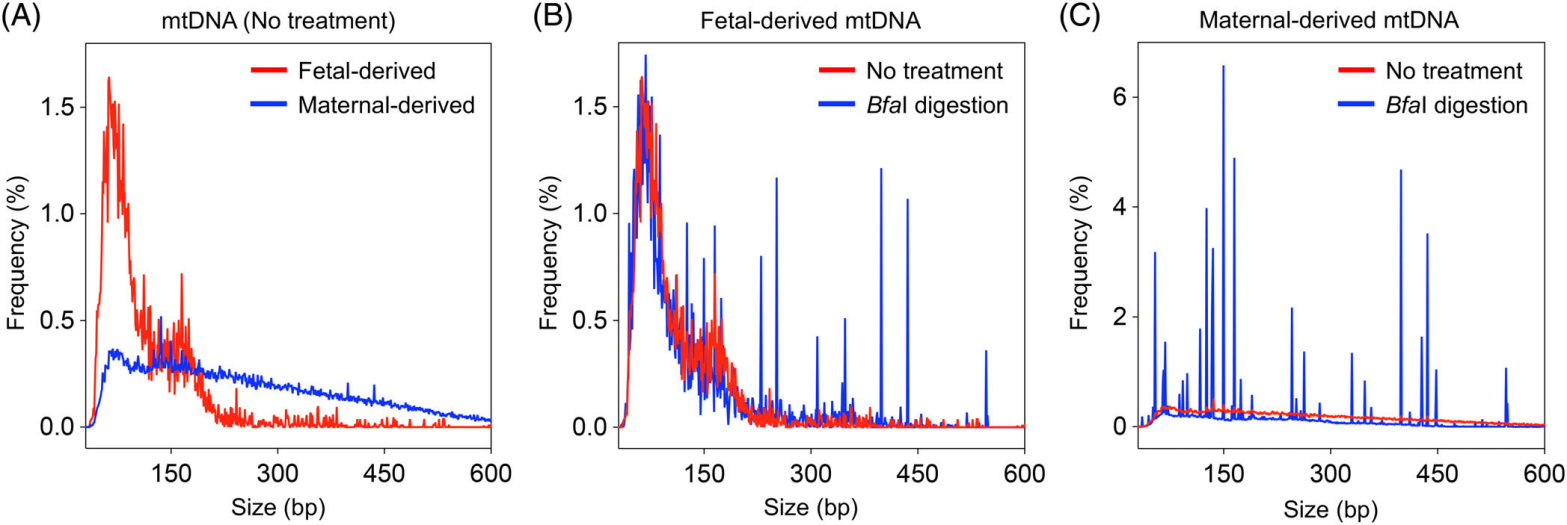
Size profiles for mtDNA molecules of fetal‐ or maternal‐derived in plasma of surrogate pregnant women. (A) Size distributions for fetal‐ and maternal‐derived mtDNA molecules in the plasma of surrogate pregnant women without enzymatic cleavage. (B) Size distributions for fetal‐derived mtDNA molecules with and without *Bfa* I cleavage in the plasma of surrogate pregnant women. (C) Size distributions for maternal‐derived mtDNA molecules with and without *Bfa* I cleavage in the plasma of surrogate pregnant women [Colour figure can be viewed at wileyonlinelibrary.com]

We next analyzed the size profiles of the linear mtDNA molecules in plasma DNA of surrogate pregnant women without *Bfa*I digestion. The fetal‐derived mtDNA molecules were much shorter than the maternal counterparts (Figure 5(A) and Figure S2). The fetal‐derived nuclear DNA fragments in all five surrogate maternal plasma DNA were found to be shorter than the maternal nuclear DNA (Figure S3), showing a 166‐bp peak with a series of 10‐bp periodic peaks in small sizes that were not observable in circulating fetal and maternal mtDNA. Such characteristic sizes of fetal and maternal nuclear DNA results were highly consistent with the previous reports.^1^, ^2^ Interestingly, the size difference between fetal and maternal mtDNA appeared to be much sharper than counterparts of nuclear DNA, as evinced by the fact that there was a 44% increase in fetal mtDNA size <100 bp but only a 1.1% increase in fetal nuclear DNA compared with corresponding maternal results, respectively.

Intriguingly, by looking at plasma mtDNA results pooled from five surrogate subjects after *Bfa*I digestion (Figure 5(B)), we observed that there were multiple discrete sporadic spikes present in the fetal mtDNA size profile, but no such spiked peaks in those samples without *Bfa*I digestion. These results suggested that the *Bfa*I cutting acted on the fetal circular mitochondrial genome in plasma DNA of surrogate pregnant women. As expected, we observed much more and more prominent spikes in the maternal mtDNA size profile, which was in line with the fact that a higher abundance of circular mtDNA was present in the maternal background DNA (Figure 5(C)).

## 4 DISCUSSION

In the present study, we showed for the first time the presence of fetal mtDNA in surrogate maternal plasma. We further investigated the linear and circular forms of mtDNA between fetal and maternal DNA, by taking advantage of the genotypic differences between fetal and maternal mitochondrial genomes in the surrogate pregnant women. Our data revealed that the linear and circular mtDNA coexisted in the plasma DNA of surrogate pregnant subjects. The fetal‐derived mtDNA in plasma was shown to be predominantly of a linear form (88%), which is reminiscent of the preponderance of linear liver‐derived mtDNA in the plasma of liver transplant recipients.^14^ In contrast, half of surrogate maternal background mtDNA (mainly of hematopoietic and liver origin) appeared to be of circular origin. The abundance of circular mtDNA in the surrogate maternal background mtDNA (51%) was lower than that reported in hematopoietically derived mtDNA (86%) in plasma of liver transplant patients.^14^ One possible explanation is that the hematopoietic cells mainly contribute circular mtDNA into plasma, whereas the liver tissues containing thousands of copies of mitochondrial genome per cell^20^ are considered as one major contributor of linear mtDNA in plasma DNA pool (74%).^14^ Therefore, the liver tissues are believed to release linear mtDNA into plasma DNA of surrogate pregnant women, thus diluting the circular mtDNA in the maternal background DNA.

However, we could not completely rule out the possibility that fragments carrying two *Bfa*I sites were originated from long linear mtDNA. According to our previous study, there was a median of 18% of pre‐existing linear mtDNA carrying at least two *Bfa*I‐cleavage sites.^14^ However, 50% of mtDNA molecules in plasma mtDNA were found to carry two *Bfa*I sites after digestion, suggesting that the circular mtDNA molecules were present in the surrogate maternal plasma DNA.

In plasma DNA without *Bfa*I digestion, the mtDNA being analyzed are likely to be spontaneously‐fragmented linear molecules, as sequencing adaptors could not be ligated to circular DNA molecules. Among the linear mtDNA molecules, the fetal mtDNA fraction was found to be relatively lower in surrogate maternal plasma compared with the fetal contribution in nuclear DNA fragments, which was opposite to the observation that liver DNA contribution was greatly enriched in linear mtDNA molecules.^14^ One possible reason would be attributed to the difference between the fetal mtDNA fraction and fetal nuclear DNA fraction. The placental cells contain lower mtDNA content^21^ than the liver cells.^20^ Thus, more linear mtDNA shed from liver tissues would dilute the placental‐derived linear mtDNA contribution.

It is surprising to see that *Bfa*I cutting patterns, originating from complete circular mitochondrial genomes, was present in fetal mtDNA size profile. These suggested the potential existence of complete fetal mitochondrial genomes in plasma. As the clearance of fetal DNA was reported to be very fast (a mean half‐life in the order of tens of minutes^22^, ^23^), the complete circular fetal mtDNA molecules might be protected from clearance by mtDNA binding proteins like mitochondrial transcription factor A (TFAM),^24^ or organelles like mitochondria and platelet.^25^ The mechanism of tissue‐specific complete circular mtDNA resistant to degradation would require further investigation.

A recent study revealed the presence of fetal and maternal extrachromosomal circular DNA (eccDNA, mainly <600 bp) in the plasma of pregnant women, showing a bimodal size distribution peaking at ~202 and ~338 bp with sharp 10‐bp periodicities.^15^ These results suggest that restriction enzyme‐based topologic analysis is not only suited for eccDNA identification but can also be used to infer the large circular DNA such as an entire circular mitochondrial genome. It would be interesting to use this approach to study whether circular DNA molecules might be associated with diseases such as cancer in the future study.

We have shown that placental mtDNA molecules are present at readily detectable amounts in maternal plasma and that they are mainly linear in nature. We believe that these realizations may catalyze the development of placental‐specific mtDNA assays even in non‐surrogate pregnancies. For example, one may focus on enriching the linear mtDNA molecules. Our research group is currently studying many other fragmentation features of cell‐free fetal DNA.^2^, ^26^, ^27^ Some of these features are more pronounced among the fetal nuclear DNA than the maternal nuclear DNA. If such additional features are identified for placental mtDNA, even more specific assays could be developed in conjunction with the findings of the present study. It needs to be noted that, in the present study, we tested surrogate pregnancies during the second and third trimester (21–31 weeks) of pregnancy. It would be desirable to analyze the plasma DNA collected from subjects during earlier pregnancy in the future. By understanding the physical features of placental mtDNA we may one day be able to detect and confirm the success, or otherwise, of mitochondrial replacement therapy (MRT)^28^, ^29^ through the analysis of placental mtDNA in the plasma samples of pregnant women whose embryos has undergone MRT.

In summary, studying surrogate pregnancies, we have demonstrated fetal mtDNA is present in the plasma of pregnant women. This observation may have clinical applications for noninvasive prenatal testing in the follow‐up of pregnancies conceived following MRT. Topologic analysis of mtDNA represents an emerging direction in plasma DNA research and may have applications beyond pregnancies to fields including cancer liquid biopsy and transplantation monitoring.

## CONFLICT OF INTEREST

K. C. Allen Chan, Rossa W.K. Chiu, and Y.M. Dennis Lo hold equities in DRA, Take2 Holdings and Grail. K.C. Allen Chan, Rossa W.K. Chiu, and Y.M. Dennis Lo are consultants to Grail. Y.M. Dennis Lo is a scientific co‐founder of Grail and serves on the scientific advisory board of Grail. Peiyong Jiang hold equities in Grail. Rossa W.K. Chiu is a consultant to Illumina. Peiyong Jiang is a director of KingMed Future. Haiqiang Zhang, Suk Hang Cheng, Peiyong Jiang, K.C. Allen Chan, Rossa W.K. Chiu, Y.M. Dennis Lo have filed a number of patent applications on cell‐free DNA based molecular diagnostics, including the technology present in this work. Patent royalties are received from Grail, Illumina, Sequenom, DRA, Take2 Health and Xcelom.

## Supporting information


**Figure S1.** Size profiles for nuclear DNA and mtDNA molecules with and without *Bfa*I digestion in plasma of surrogate pregnant women. (A) Size profiles of mtDNA fragments in surrogate maternal plasma DNA with and without *Bfa*I digestion. (B) Size profiles of nuclear DNA fragments in surrogate maternal plasma DNA with and without *Bfa*I digestion.

**Figure S2.** Plots of size profiles for fetal‐ and maternal‐derived mtDNA molecules in plasma of five surrogate pregnant women (SP1‐5).

**Figure S3.** Plots of size profiles for fetal‐ and maternal‐derived nuclear DNA molecules in plasma of five surrogate pregnant women (SP1‐5).

## Data Availability

Research methods are available by contacting the corresponding author. Participants have not consented for sequence data sharing.
