# Supplementary figures and images for: Fetal mitochondrial DNA in maternal plasma in surrogate pregnancies: Detection and topology
Source: Prenat Diagn. 2020 Nov 12;41(3):368–75. doi: 10.1002/pd.5860 (PMC7984455; doi:10.1002/pd.5860)

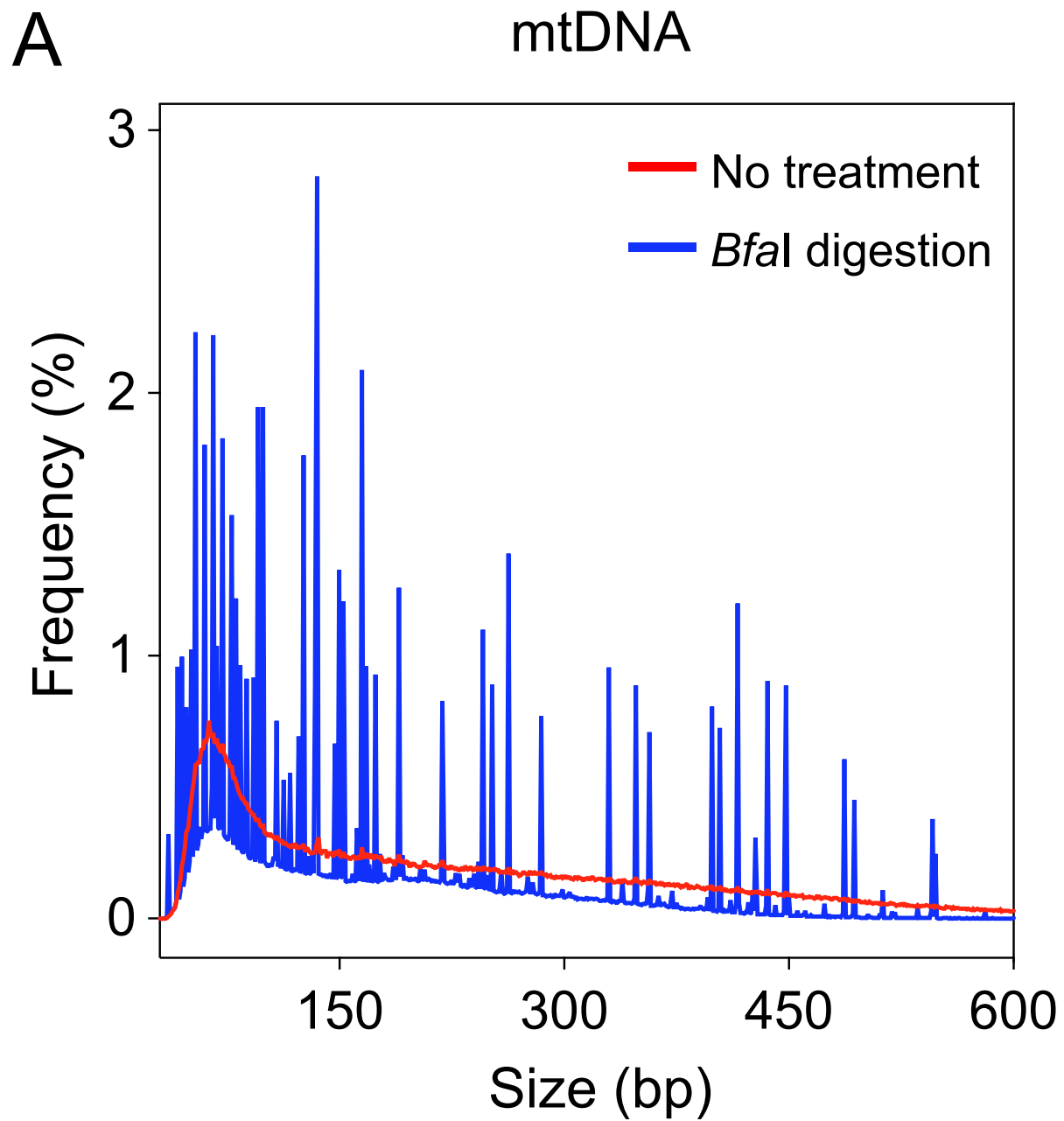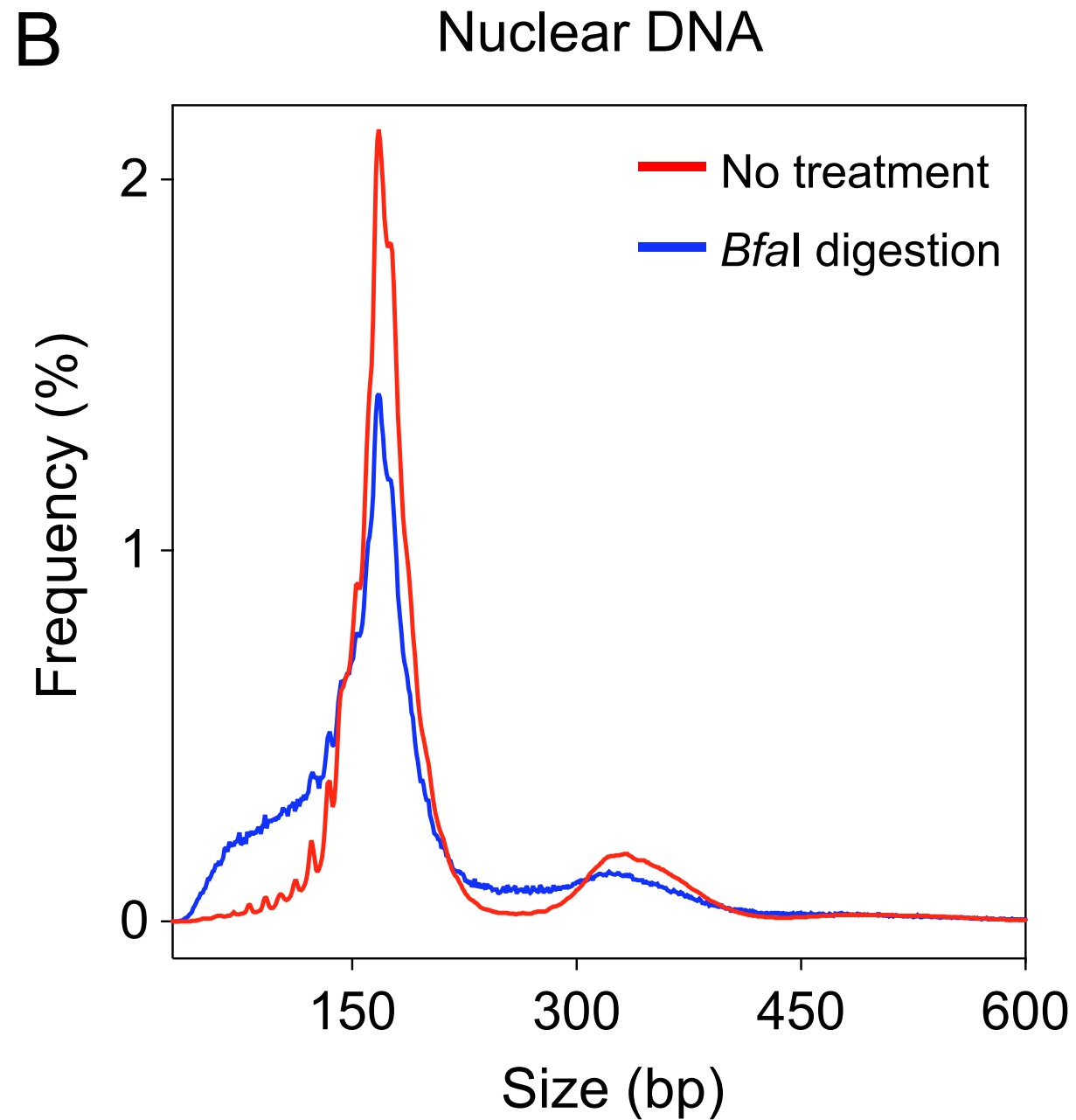

Figure S1

Supplement: Supplementary file 1 — Figure S1. Size profiles for nuclear DNA and mtDNA molecules with and without BfaI digestion in plasma of surrogate pregnant women. (A) Size profiles of mtDNA fragments in surrogate maternal plasma DNA with and without BfaI digestion. (B) Size profiles of nuclear DNA fragments in surrogate maternal plasma DNA with and without BfaI digestion. [file PD-41-368-s002.pdf]

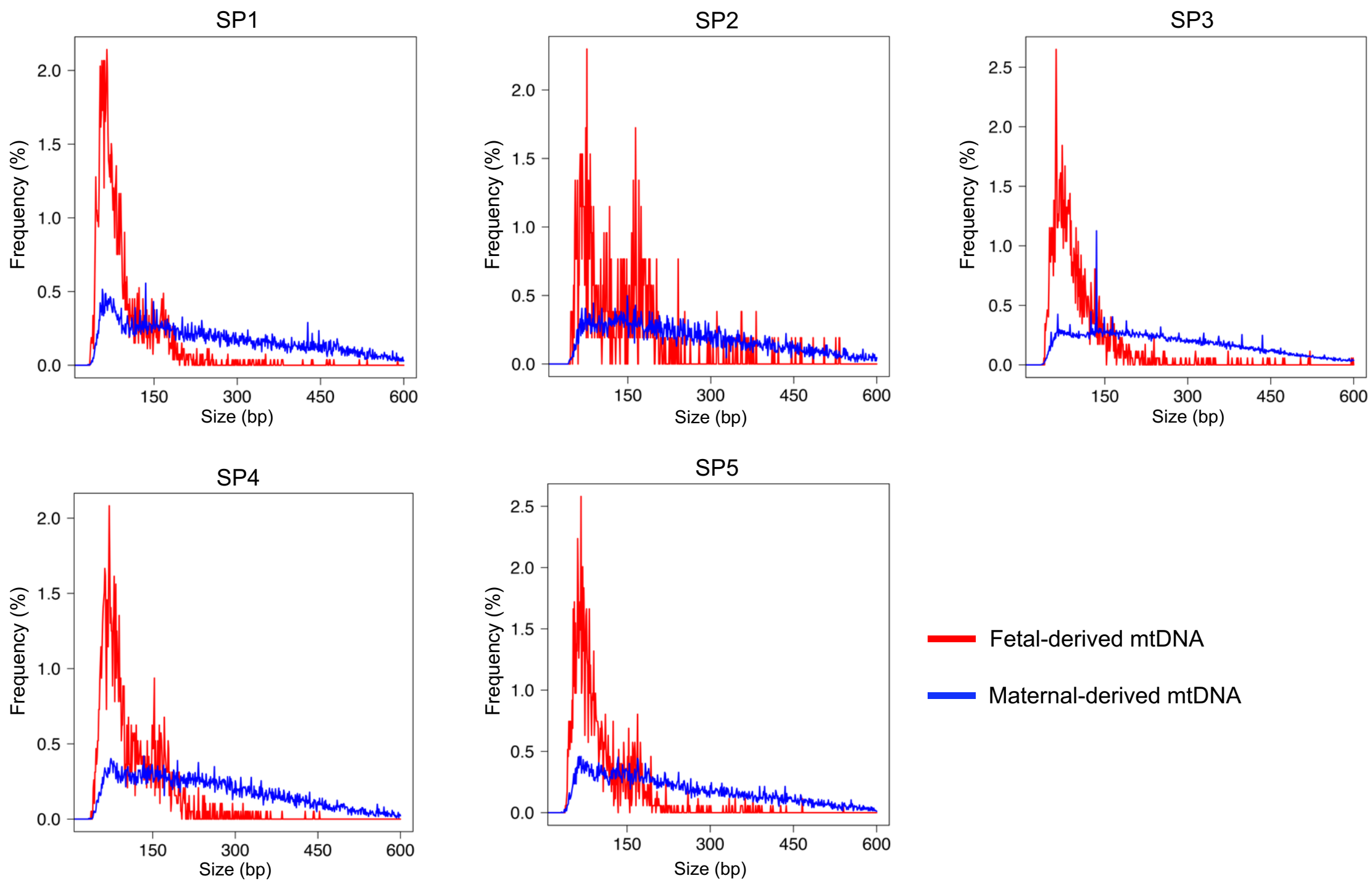

Figure S2

Supplement: Supplementary file 2 — Figure S2. Plots of size profiles for fetal‐ and maternal‐derived mtDNA molecules in plasma of five surrogate pregnant women (SP1‐5). [file PD-41-368-s001.pdf]

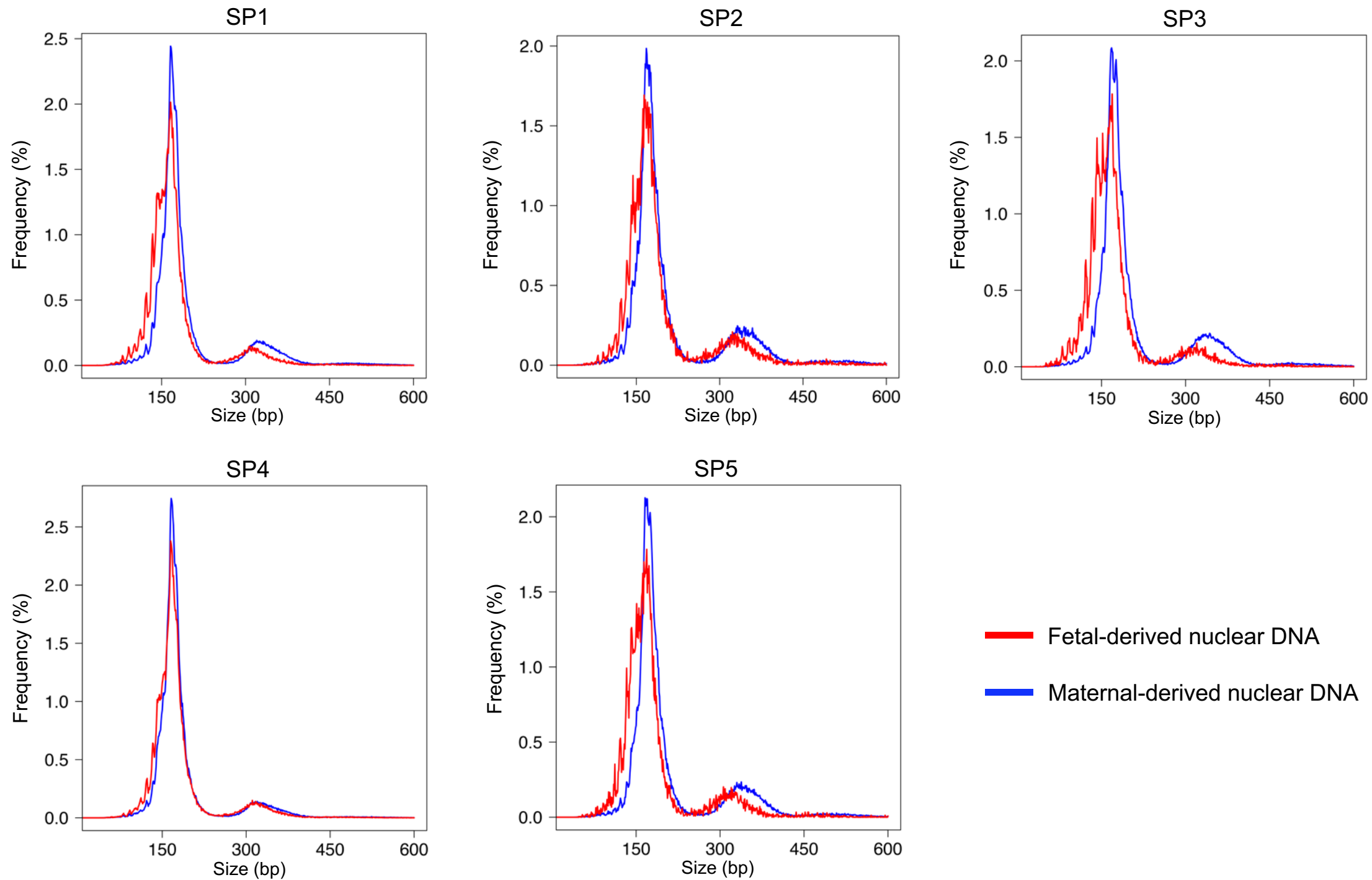

Figure S3

Supplement: Supplementary file 3 — Figure S3. Plots of size profiles for fetal‐ and maternal‐derived nuclear DNA molecules in plasma of five surrogate pregnant women (SP1‐5). [file PD-41-368-s003.pdf]
